# German Medical Named Entity Recognition Model and Data Set Creation Using Machine Translation and Word Alignment: Algorithm Development and Validation

**DOI:** 10.2196/39077

**Published:** 2023-02-28

**Authors:** Johann Frei, Frank Kramer

**Affiliations:** 1 IT Infrastructure for Translational Medical Research University of Augsburg Augsburg Germany

**Keywords:** natural language processing, named entity recognition, information extraction

## Abstract

**Background:**

Data mining in the field of medical data analysis often needs to rely solely on the processing of unstructured data to retrieve relevant data. For German natural language processing, few open medical neural named entity recognition (NER) models have been published before this work. A major issue can be attributed to the lack of German training data.

**Objective:**

We developed a synthetic data set and a novel German medical NER model for public access to demonstrate the feasibility of our approach. In order to bypass legal restrictions due to potential data leaks through model analysis, we did not make use of internal, proprietary data sets, which is a frequent veto factor for data set publication.

**Methods:**

The underlying German data set was retrieved by translation and word alignment of a public English data set. The data set served as a foundation for model training and evaluation. For demonstration purposes, our NER model follows a simple network architecture that is designed for low computational requirements.

**Results:**

The obtained data set consisted of 8599 sentences including 30,233 annotations. The model achieved a class frequency–averaged *F*_1_ score of 0.82 on the test set after training across 7 different NER types. Artifacts in the synthesized data set with regard to translation and alignment induced by the proposed method were exposed. The annotation performance was evaluated on an external data set and measured in comparison with an existing baseline model that has been trained on a dedicated German data set in a traditional fashion. We discussed the drop in annotation performance on an external data set for our simple NER model. Our model is publicly available.

**Conclusions:**

We demonstrated the feasibility of obtaining a data set and training a German medical NER model by the exclusive use of public training data through our suggested method. The discussion on the limitations of our approach includes ways to further mitigate remaining problems in future work.

## Introduction

### Overview

Despite continuous efforts to transform the storage and processing of medical data in health care systems into a framework of machine-readable highly structured data, implementation designs that aim to fulfill such requirements are only slowly gaining traction in the clinical health care environment. In addition to common technical challenges, physicians tend to bypass or completely avoid inconvenient data input interfaces, which enforce structured data formats, by encoding relevant information as free-form unstructured text [1,2].

Electronic data capturing systems are developed to improve the situation of structured data capturing. Yet their primary focus lies on clinical studies. The involvement of these systems needs to be designed in early stages and requires active software management and maintenance. Such electronic data capturing solutions are commonly considered in the context of clinical research but are largely omitted in non–research-centric health care services, and paper-based solutions are preferred [1-4].

Because of the rise of data mining and big data analysis, finding and understanding novel relationships of disease, disease-indicating biomarkers, drug effects, and other input variables require large-scale data acquisition and collection. This induces additional pressure on finding and exploring new possible data sources.

Although new data sets can be designed and created for specific use cases, the amount of obtained data might be very limited and not sufficient for modern data-driven methods. Furthermore, such data collection efforts can turn out as rather inefficient in terms of time and work involved in creating new data sets with respect to the number of acquired data samples.

In contrast, unstructured data of sources from legacy systems and non–research-centric health care, referred to as second use, offer a potential alternative. However, techniques for information extraction and retrieval, mainly from the natural language processing (NLP) domain, need to be applied to transform raw data into structured information.

While the availability of existing NLP models in English, and other non–NLP-based techniques, for medical use cases is the focus of active research, the situation of medical NLP models for non-English languages is less satisfying. As the performance of an NLP model often depends on its dedicated target language, most models cannot be shared and reused easily in different languages but require retraining on new data from the desired target language.

In particular, for the case of detection of entities like prescribed drugs and level or frequency of dosage from German medical documents like doctoral letters, few open and publicly available models have been published. We attribute this to two main contributing factors:

1. Lack of public German data sets: Most open public data sets are designed for English data only. Until 2020, no such dedicated German data set has been published. Specifically in the context of clinical data, legal restrictions and privacy policies prevent the collection and publication of German data sets. Data-driven NLP research for medical applications uses largely internal data for training and evaluation. In addition to the data set itself, to model relevant text features with supervised learning, high-quality annotations of the data set are essential for robust model performance.
2. Protection of sensitive data and privacy concerns: Although few works have been published that present data-driven models for German texts, the weights of these models have not been openly published. Because respective training data have been used in a nonanonymized or pseudonymized fashion, the publication of the model weights inherently comes at the risk of possible data leakage issues through training data extraction [5] from the model, potentially exposing sensitive information like patient names or ID numbers.

In this paper, we aim to tackle the scarcity issue of anonymous training data and publicly available medical German NLP models. Our main contributions are as follows:

- Automated retrieval of German data set: We propose a method to create a custom data set for our target language, based on a public English data set. In addition, we apply a strategy to preserve relevant annotation information across languages.
- Training of medical German NLP model component: We trained and built a named entity recognition (NER) component on the custom data set. The model pipeline supports multiple types of medical entities.
- Evaluation and publication of the NLP component: The retrieved data set and the NER model were evaluated as part of an NLP pipeline. The trained model is publicly available for further use by third parties.

### Related Work

In recent years, substantial progress has been made in the area of NLP, which can mostly be attributed to the joint use of large amounts of data and their processing through large language models like BERT (Bidirectional Encoder Representations from Transformers) [6] and its (bio)medical-specific models [7-12]. Such elements display a straightforward way to encode representations of semantic information for further processing in downstream tasks like text classification or text segmentation. These works mostly focus on the English language because of available language corpora like scientific texts from PubMed or specifically designed corpora such as *n2c2* [13] (with annotations) and *MIMIC-III* [14]. For German, only a few works such as *GGPONC* [15] and *BRONCO* [16] have been published in recent years as data sets that carry annotation information. Other German data sets [17,18] lack annotation information. Moreover, the *Technical-Laymen* [19] corpus provides an annotated corpus, yet it is based on crawled texts from nonprofessional online forums. Various other German medical text corpora exist [20-31] as a basis for certain NLP and information extraction use cases but are inaccessible for public distribution.

In the field of NLP systems for German medical texts, *medSynDiKATe* [32,33] approaches information extraction on pathological finding reports by parsing and mapping text elements to (semi)automatically build knowledge representation structures. Processing of pathological findings in German has also been applied to the tasks of sentence classification [22].

In the context of patient records, a hybrid relation extraction (RE) and NER parsing approach using the *SProUT* [34] parser has been proposed [35]; however, the entity tags lack medical relevance. A similar general NER for nonmedical entity tags has been applied to enable the deidentification of clinical records [36] using statistical and regex-based models through the *StanfordNLP* parser [37].

Neural methods have been shown to perform well on certain NLP tasks. In particular, convolutional neural network (CNN) approaches for RE [38-40] have become popular in recent years. For German texts, the performance of various methods has been investigated for medical NER tasks [41], such as CNN, long short-term memory, or support vector machine–based models. In this context, the text processing platform *mEx* [42] uses CNN-based methods for solving medical NER in German texts. Similar to our work, *mEx* is built on *SpaCy* [43] but provides custom models for other NLP tasks such as RE. However, the platform has been partially trained on internal clinical data, and thus, its statistical models have not been openly published and may only be used under certain legal restrictions on request. An updated version has been published [44], yet the models can only be retrieved on request under a usage agreement. As additional work, *GGPONC* (release 2.0) [45] provides a baseline model on request. For a more exhaustive survey on non-English clinical NLP in general, we point to [46].

With respect to obtaining cross-lingual annotation information, the basic concept of projecting label data in language pairs via word alignment has been discussed in various NLP contexts [47-57]. For medical use cases, little research exists [49], with focus on English and Chinese data and models. Yet German medical contexts remain largely unexplored.

## Methods

### Overview

**Figure 1:**
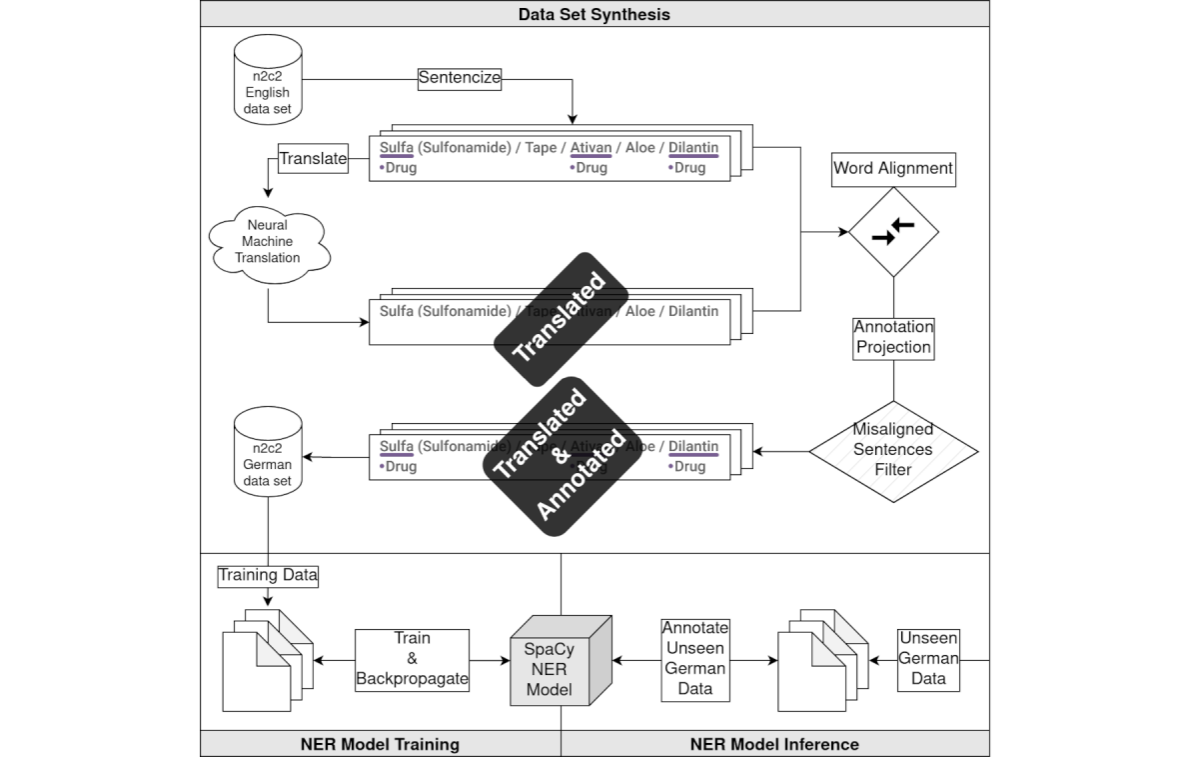
Illustration of the data set creation and natural language processing (NLP) model training process. The initial English *n2c2* data set is transformed into a synthetic German data set. The data set is used for training an NLP NER model. NER: named entity recognition.

In this section, we first describe our method to synthesize the data set and then describe the used NER model for German text tagging. The entire pipeline is illustrated in Figure 1.

### Custom Data Set Creation

We relied on the publicly available training data from the *n2c2 NLP 2018 Track 2* [13] data set (adverse drug event [ADE] and medication extraction challenge) as our initial source data set. The data were composed of 303 annotated text documents that have been post processed by the editor for anonymization purposes to explicitly mask sensitive privacy-concerning information. They featured the annotation labels *Drug*, *Route*, *Reason*, *Strength*, *Frequency*, *Duration*, *Form*, *Dosage,* and *ADE*.

To transform the data into a semantically plausible text, we identified the type and text span of text masks such that we were able to replace the text masks by sampling type-compatible data randomly from a set of sample entries. During the sampling stage, depending on the type of mask, text samples for entities like dates, names, years, or phone numbers were generated and inserted into the text. Because every replacement step might affect the location of the text annotation labels as provided by the character-wise start and stop indices, these label annotation indices must be updated accordingly. For further preprocessing, we split up the text into single sentences such that we could omit all sentences with no associated annotation labels.

For automated translation, we made use of the open source *fairseq* (version 0.10.2) [58] model architecture. *fairseq* is an implementation of a neural machine translation model that supports the automatic translation of sequential text data using pretrained models. For our purposes, we ran the *transformer.wmt19.en-de* pretrained model to translate our set of English sentences into German because the model shows a strong BLEU (BiLingual Evaluation Understudy) translation score for English-German translation tasks [59] while maintaining its simplicity for deployment.

The reconstructive mapping of the annotation labels from the English source text to the German target text was tackled by *fast_align* [60]. *fast_align* is an unsupervised method for aligning words from 2 sentences of source and target language. The choice for *fast_align* was reasoned by its low-resource footprint, and it can align sentences fast through its simple statistical model. We projected the annotation labels onto the translated German sentences using the word-level mapping between the corresponding English and German sentence to obtain new annotation label indices in the German sentence. In nonmedical contexts, similar work on non-German target languages exists (eg, [57]).

The word alignment mapping tends to induce errors in situations of sentences with irregular structures such as tabular or itemized text sections. We mitigated the issue and potential subsequent error propagation by inspecting the structure of the word mapping matrix *A*:



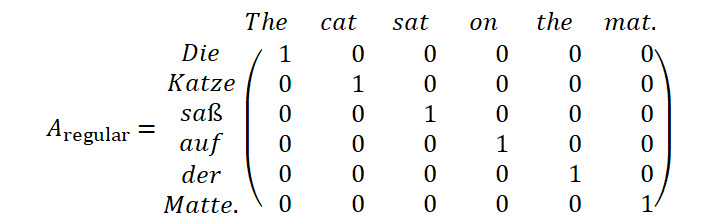



In situations where *fast_align* fails to establish a meaningful mapping between the source and target sentence, it can be observed that the resulting mapping table collapses to a highly nondiagonal matrix structure, as illustrated by the following example:



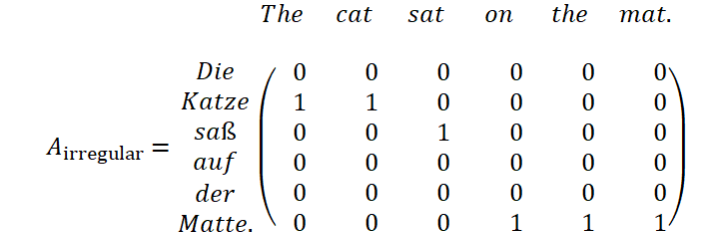



Severely ill-aligned word mapping matrices can be detected and removed from the final set of sentences by applying the simple filter decision rule:



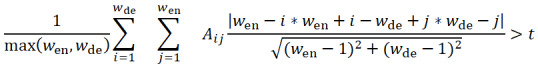



where the average distance between a nonzero entry and the diagonal line from *A*_1,1_ to 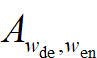 is evaluated, given *w_en_* as the number of words in the English sentence and *w_de_* as the number of words in the German sentence. If the value exceeds the threshold *t*, the sentence pair is disregarded for the final set of sentences.

The word mapping matrices describe a nonsymmetric cross-correspondence between 2 language-dependent token sets, which enables the projection of tokens within the English annotation span onto the semantically corresponding tokens in the German translation text. Therefore, the annotation label indices for the English text can be resolved to the actual indices for the translated German text at a character level.

### NER Model Architecture

For the buildup of our NER model as part of an NLP pipeline, we use *SpaCy* as an NLP framework for training and inference. In comparison with state-of-the-art models, we select a lightweight non–transformer-based model because it serves primarily as a demonstration model and can be trained without significant compute costs.

- Embedding: The word tokens are embedded by Bloom embeddings [61] where different linguistic features are concatenated into a single vector and passed through *n*_embed_ separate dense layers, followed by a final max pooling and layer norm step. This step enables the model to learn meaningful linear combinations of single input feature embeddings while reducing the number of dimensions.
- Context-aware token encoding: To extract context-aware features that are able to capture larger token window sizes, the final token embedding is passed through a multilayered convolutional network. Each convolution step consists of the convolution itself and the following max-pooling operation to keep the dimensions constrained. For each convolution step, a residual (skip) connection is added to allow the model to pass intermediate data representations from previous layers to subsequent layers.
- NER parsing: For each encoded token, a corresponding feature-token vector is precomputed in advance by a dense layer. For parsing, the document is processed token-wise in a stateful manner. For NER, the state at a given position consists of the current token, the first token of the last entity, and the previous token by index. Given the state, the feature-position vectors are retrieved by indexing the values from the precomputed data and summed up. A dense layer is applied to predict the next action. Depending on the action, the current token is annotated and the next state is generated until the entire document has been parsed.

### Ethical Considerations

Because of the nature of our proposed method, our work does not involve data or human subject research, which could potentially violate basic human ethics in a narrow sense.

The public data approach shifts the responsibility of privacy-preserving measures to the data set publisher. We assume that the *n2c2* data set has been deidentified correctly and no privacy-related information can be retrieved anymore.

## Results

### Data Set Synthesis

The source data set consists of 303 documents from the *n2c2* training data set. As an initial preprocessing step, we needed to replace the anonymization masks with meaningful regular text data to reconstruct the natural appearance of the text and alleviate a potential data set bias that leads to gaps between the data set and real-world data. For numerical data, we could retrieve mask replacements by random sampling. Similar to numerical data, dates and years are sampled and formatted to common date formats. For semantically relevant data types, we used the Python package *Faker*. The package maintains lists of plausible data of various types such as first names, last names, addresses, or phone numbers. We made use of these data entries for certain types of anonymization masks.

To obtain our custom data set, we split the texts from the original data set into single sentences using the sentence splitting algorithm from *SpaCy*. The English sentences were translated into German by the *fairseq* library with beam search (*b*=5). The sentence-wise word alignments were obtained by *fast_align* and cleaned up by our filter decision rule (*t*=1.8). To determine this particular hyperparameter, we sampled 10 ill-aligned samples without applying the filter and gradually lowered the threshold *t* until all 10 samples were detected by the decision rule.

The labels *Reason* and *ADE* were removed from the data set because of the fact that their definitions are rather ambiguous in general contexts beyond the scope of the initial source data set.

Our final custom data set consisted of 8599 sentence pairs, annotated with 30,233 annotations of 7 different class labels. The different class labels and their corresponding frequency in absolute numbers are shown in Table 1. The German sentences consisted of 172,695 tokens in total.

**Table 1:** The model performance scores per named entity recognition (NER) tag and the annotation distribution in the custom data set in absolute numbers.^a^

NER tag	Precision (%)	Recall (%)	*F*_1_ score (%)	Label tags,n
Drug	67.33	66.17	66.74	8305
Strength	92.34	90.99	91.66	4071
Route	89.93	90.14	90.04	4549
Form	91.94	89.24	90.57	4238
Dosage	87.83	87.57	87.70	409
Frequency	79.14	76.92	78.01	5242
Duration	67.86	52.78	59.37	3419
Total	82.31	80.79	81.54	30,233

^a^The evaluation is based on the separated test set. Total scores are aggregated by label-frequency-weighted averaging. The total data set consists of 8599 sentence samples (172,695 tokens). A single-tag sample may span multiple tokens.

### Translation and Alignment Artifacts

**Figure 2:**
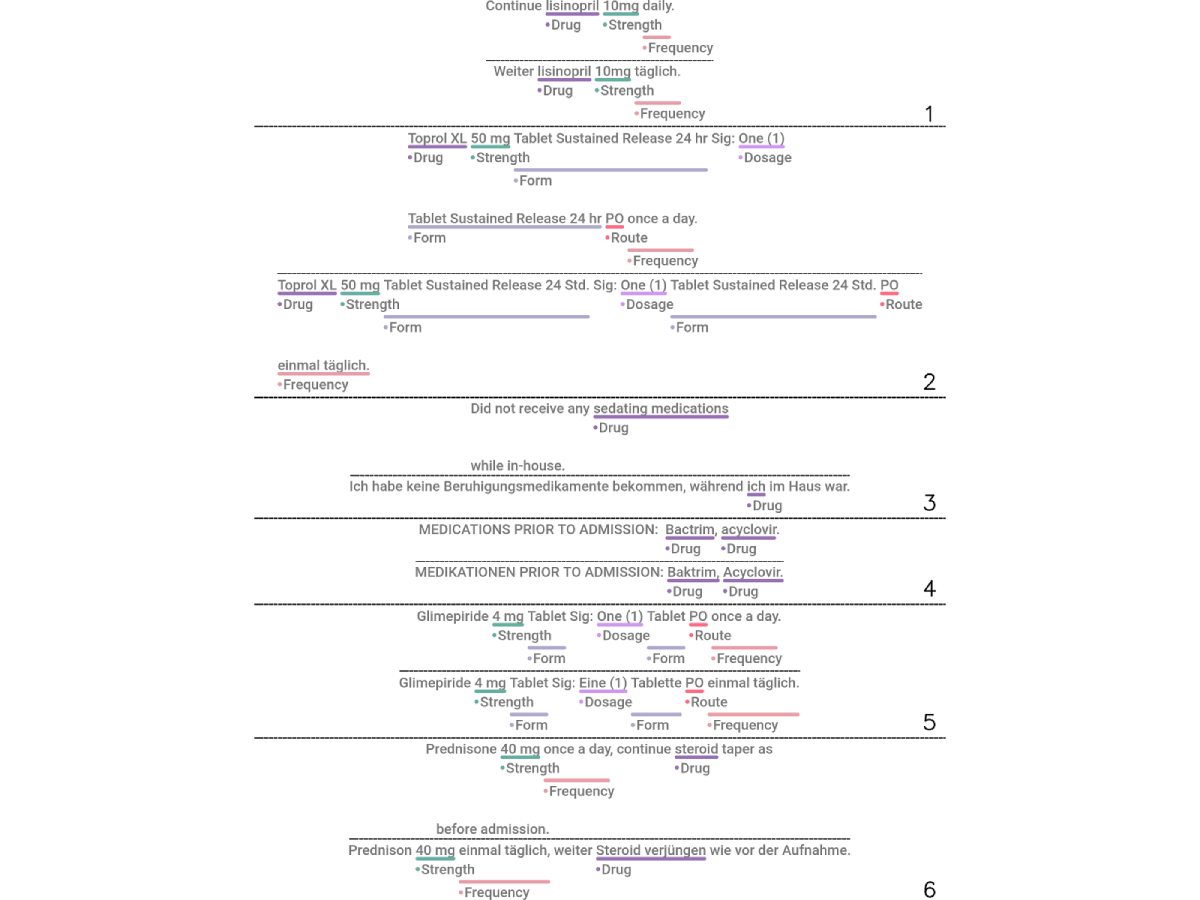
Selection of sampled sentence pairs from the synthetic data set. Most samples show correct outputs. The translation and word alignment artifacts occur in unusual syntactical contexts (translation) or complex sentence structures (alignment). Both English sentence (top) and its translated sentence (bottom) are depicted. Annotations with failed German correspondence resolution are not shown in the English sentence.

We sampled and selected a set of sentence pairs to investigate and illustrate the artifacts that we could observe in the synthesized data set with regard to translation as well as word alignment. The selection of samples is presented in Figure 2. Overall, we found the alignment and translation quality acceptable in sentences of simple structure and semantics (sample 1). However, the translation tended to fail in abbreviations such as PO (samples 2 and 5) as well as in text with uncommon syntax such as uppercase text (sample 4) or domain-dependent context (samples 2 and 6). To our surprise, the translation model was able to translate the sequence “One (1)” correctly in sample 5 but failed for the same term in sample 2. We attribute this to the context-sensitive, neural black-box model of the translation engine. In terms of alignment, most tokens were well aligned in sentence pairs of simple syntax and structure. Alignment errors could be found in sentence pairs with different sentence structures in English and German (sample 3) where our filter rule does not apply.

Because of parsing and alignment issues, we found that annotations were discarded (samples 5 and 6) in cases of single-token annotations that start as the first word of the sentence. This artifact affected primarily the label class *Drug*.

### NER Model Training and Evaluation

For training, we used our custom German data set as our training data and split the data set into a training set (80%, 6879 sentence samples), validation set, and test set (both 10%, 860 samples). The training setup followed the default NER setup of *SpaCy*; the Adam optimizer with a learning rate of 0.001 with decay (β_1_=.9, β_2_=.999) was used. The training took 10 minutes on an Intel i7-8665U CPU.

**Figure 3:**
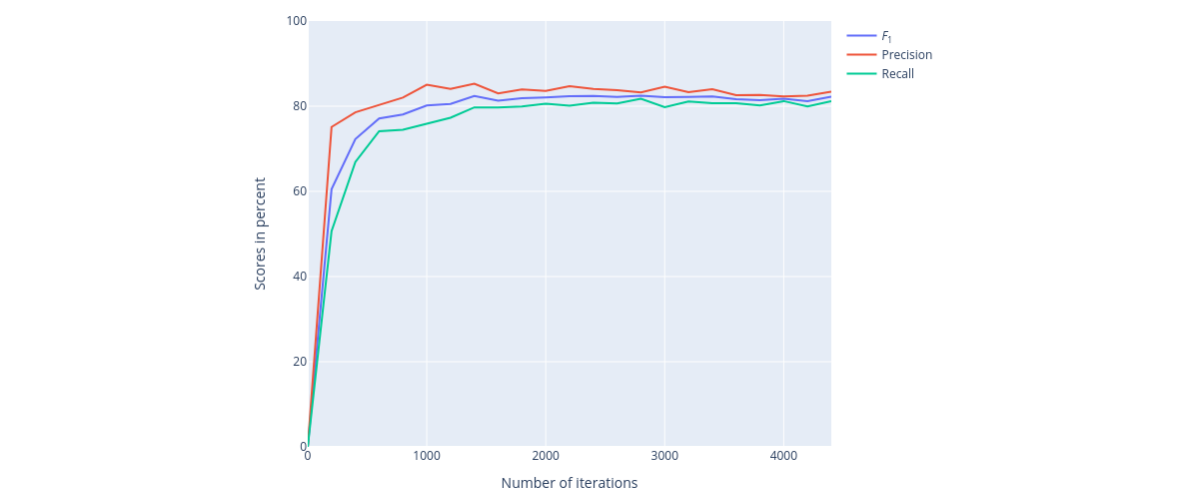
Training scores on validation set (as part of the training set): evaluation scores are computed at every 200th iteration.

The model performance during training is shown in Figure 3. The corresponding performance scores were evaluated on the validation set (as part of the training set).

We selected the final model based on the highest *F*_1_ score on the validation set. The performance of the selected model was evaluated on the test set per NER tag as well as in total. The evaluation concerns the token-wise IOB (Inside, Outside, Begin)-action prediction. The results are shown in Table 1.

### Comparison to the English Baseline Data Set

To empirically quantify the error propagation through translation and word alignment, we retrained an equivalent model with all English sentences from our sentence pairs. The evaluation strategy remained similar to the strategy for the scores from Table 1. The scores are reported in Table 2. The results from the English model show comparable results to the German model for all labels except for *Drug*.

**Table 2:** Test set performance scores per named entity recognition (NER) tag of the model trained on the English sentences from the obtained data set in absolute numbers.^a^

NER tag	Precision (%)	Recall (%)	*F*_1_ score (%)	German *F*_1_ score (%)
Drug	80.94	82.02	81.47	−14.73 (66.74)
Strength	89.02	90.12	89.57	2.09 (91.66)
Route	85.55	95.08	90.06	−0.02 (90.04)
Form	94.36	87.12	90.60	−0.03 (90.57)
Dosage	89.41	89.97	89.69	−1.99 (87.70)
Frequency	80.55	80.15	80.35	−2.34 (78.01)
Duration	62.50	51.02	56.18	3.19 (59.37)
Total	85.14	85.82	85.48	−3.94 (81.54)

^a^The evaluation similar to the results from Table 1. Total scores are aggregated by label-frequency-weighted averaging. For comparison, the *F*_1_ score differences of the German model to the English model are provided.

### Advanced Evaluation and Model Comparison on a Separated Data Set

To further estimate the performance scores on a separated data set, we evaluated the model on a custom out-of-distribution (OoD) data set. The data set was created internally by clinical physicians by manually writing down and annotating 30 fake sentences (*Internal Gold*). For model comparison, we used the baseline model from *GGPONC* (release 2.0) [45] and evaluated its annotation performance on the label class we considered equivalent to our *Drug* label class. In comparison with our model (approximately 5 MB), the *GGPONC* model is orders of magnitudes larger due to its use of pretrained transformers (approximately 500 MB). The results are given in Table 3. The *F*_1_ score corresponds to the character-wise label classification performance.

**Table 3:** Evaluation on our out-of-distribution data set with the related GGPONC baseline model for reference: the model performance drops significantly for certain infrequent label classes.^a^

Data set and GERNERMED		Sample, n	*F*_1_ score (%)	*GGPONC* baseline	F_1_ score (%)
**Internal Gold (30 sentences)**					
	Drug	36	54.48	Chemicals_Drug	56.07
	Strength	37	67.70	No equivalent	N/A^b^
	Form	19	23.83	No equivalent	N/A
	Dosage	4	02.47	No equivalent	N/A
	Frequency	20	48.14	No equivalent	N/A
	Duration	3	0	No equivalent	N/A

^a^The *F*_1_ scores are evaluated as performance scores of character-wise label classifications. The label classes Dosage and Duration occur less frequently and therefore their scores are less reliable.

^b^N/A: not applicable.

## Discussion

### Principal Findings

We were able to obtain a synthetic German data set for medical purposes from an English data set by the method proposed in previous sections. As expected, the translation and alignment method introduced artifacts into the output data, but our model was still able to yield proper performance on the test set after training on the training set from our synthetic data set.

Separate training on the English sentence pairs yielded similar results for all label classes except for the *Drug* label class. Because it can be assumed that the inherent structure and vocabulary bias from the data set are preserved through translation, the drop for *Drug* can be explained by 2 joint reasons. First, the lexical properties of a translated *Drug* word to its source word can differ frequently and severely. More basic tokens like from the label *Strength* lack language-dependent elements and can be aligned in a robust manner. In cases of other label classes, phrases are often less diverse or are used repeatedly because of the data set bias. This enables robust alignments from *fast_align* due to their statistically frequent correlations.

When evaluating and comparing our model with another model on an OoD data set, we observed a drop in performance scores across labels. Aside from the label classes of low sample size, we attributed the gaps to the data set shift, which was not captured well by the underlying model architecture. The model cannot rely on high-level semantic embeddings but relies on basic structural patterns, and thus, it works well on the test set but yields less accurate results on independent data sets. The model is intentionally kept primitive as it is meant to serve as a demonstration of feasibility and does not make use of a pretrained transformer.

### Limitations

Because non–drug-related label classes are not available as annotation data in most external data sets, we cannot independently quantify the drop in performance on these label classes. In the context of this work, it further remains unclear how impactful the use of pretrained transformer networks will be in terms of annotation performance on external data sets if it is trained on the synthesized data set. In this work, the choice of the statistical model and the slim neural model architecture, in particular, is attributed to its small computational footprint while being able to achieve satisfying results. In addition, the NER pipeline of *SpaCy* explicitly induces inductive bias through hand-crafted feature extraction during the token embedding stage. However, the focus of our work lies on the presentation of the translation and alignment method for data set synthesis and its demonstration data for training purposes in the German medical context. We consider an exhaustive hyperparameter optimization as well as the use of a transformer-based model as future work.

In general, the availability of German NER models and methods for medical and clinical domains still leaves much to be desired as described in previous sections. German data sets in this domain have been largely kept unpublished in the past. However, its implications are significantly broader. In the case of unpublished NLP models, it renders independent reproduction of results and fair comparisons impossible. In the case of lacking data sets or inconsistent annotations, novel competitive data-driven techniques cannot be developed or validated easily.

### Conclusions

In this paper, we presented our method for obtaining a synthesized data set and neural NER model for German medical text as an open, publicly available model. We trained the model on our custom German data set from a publicly available English data set. We described the method to extract and postprocess texts from the masked English texts and generate German texts by translating and cross-lingual token aligning. In addition, the NER model architecture was described and the final model performance was evaluated for single NER tags as well as its performance in total. We discussed the observed issues with the synthesized data set and the performance drop through data set shifts. The advanced evaluation was done on an independent OoD data set. We believe that our method is a well-suited foundation for future work in the context of German medical entity recognition and natural language processing. In particular, the use of primitive NER model architecture remains an important point for future work. The need for independent data sets to further improve the situation for the research community on this matter has been highlighted.

The model and the test set corpus are available on GitHub [62].

## Abbreviations

ADE: adverse drug event
CNN: convolutional neural network
NER: named entity recognition
NLP: natural language processing
OoD: out-of-distribution
RE: relation extraction
